# Human and financial resource needs for universal access to WHO-PEN interventions for diabetes and hypertension care in Eswatini: results from a time-and-motion and bottom-up costing study

**DOI:** 10.1186/s12960-024-00913-0

**Published:** 2024-05-27

**Authors:** Harsh Vivek Harkare, Brianna Osetinsky, Ntombifuthi Ginindza, Bongekile Thobekile Cindzi, Nomfundo Mncina, Babatunde Akomolafe, Lisa-Rufaro Marowa, Nyasatu Ntshalintshali, Fabrizio Tediosi

**Affiliations:** 1 Swiss Tropical and Public Health Institute, Kreuzstrasse 2, 4123 Allschwil, Switzerland; 2 University of Basel, Basel, Switzerland; 3 Ministry of Health in Eswatini, Ministry of Justice & Constitutional Affairs Building, Mhlambanyatsi Road, Mbabane, Eswatini; 4 Clinton Health Access Initiative, Mbhilibhi House, Plot 170, Corner Tsekwane/Mbhilibhi Street, Mbabane, Eswatini

**Keywords:** Costing, Human resources, Time-and-motion, Diabetes, Hypertension, Eswatini, WHO-PEN, Differentiated service delivery, Community outreach

## Abstract

**Background:**

Eswatini faces persistent challenges in providing care for diabetes and hypertension, exacerbated by a shortage of healthcare workers. The implementation of WHO-PEN interventions aimed to address these issues, yet their effects on healthcare worker time requirements and associated costs remain unclear.

**Methods:**

This study employed a time-and-motion analysis and a bottom-up cost assessment to quantify the human and financial resources required for scaling up WHO-PEN interventions nationally in Eswatini for all estimated diabetic and hypertensive patients.

**Results:**

Findings reveal that healthcare workers in intervention-arm clinics reported longer workday durations compared to those in control-arm clinics, yet spent less time per patient while seeing more patients. The implementation of WHO-PEN interventions increased the workload on healthcare workers but also led to a notable increase in patient care utilization. Furthermore, a morning peak in patient visits was identified, suggesting potential opportunities for optimizing patient flow. Notably, scaling up care provision nationally with WHO-PEN interventions proved to be more cost saving than expanding standard-of-care treatment.

**Conclusion:**

WHO-PEN interventions hold promise in improving access to diabetes and hypertension care in Eswatini while offering an efficient solution. However, addressing challenges in healthcare workforce creation and retention is crucial for sustained effectiveness. Policy makers must consider all aspects of the WHO-PEN intervention for informed decision-making.

*Trial registration* US Clinical Trials Registry. NCT04183413. Trial registration date: December 3, 2019. https://ichgcp.net/clinical-trials-registry/NCT04183413

**Supplementary Information:**

The online version contains supplementary material available at 10.1186/s12960-024-00913-0.

## Background

The increasing prevalence of non-communicable diseases (NCDs), notably diabetes mellitus (DM) and hypertension (HTN), presents a burgeoning public health crisis in the Kingdom of Eswatini, located in Southern Africa. Estimates of disease burden vary widely, with figures suggesting that between 25 to 60% of individuals aged 40 and above suffer from some degree of diabetes or hypertension [3, 17, 18]. Compounding this issue, Eswatini, akin to many sub-Saharan African (SSA) nations, grapples with a critical shortage of healthcare professionals [16]. Access to primary healthcare clinics (PHCs) remains a significant challenge, particularly for rural populations [13].

Recognizing the barriers to care for DM/HTN, the Ministry of Health in Eswatini has identified several challenges, including the concentration of disease management in urban tertiary care clinics and hospitals [15]. In response to the COVID-19 pandemic and its strain on tertiary healthcare facilities, the Government of Eswatini decentralized diabetes and hypertension care to PHCs. Furthermore, the economic burden of managing diabetes and hypertension affects both patients and the healthcare systems that provide their care. While patient costs have been extensively documented [14], the financial implications for health systems and healthcare providers remain less understood, particularly within the African context [1, 6, 11].

As part of the WHO-PEN@Scale consortium, we implemented the World Health Organization (WHO) Package of Essential Non-Communicable Disease (NCD) (WHO-PEN) interventions in Eswatini to enhance access to diabetes and hypertension care. While WHO-PEN interventions are anticipated to be cost-effective, the decentralization of care and the projected surge in patient volume due to improved accessibility are expected to strain an already burdened healthcare system. Accurately quantifying this strain is essential for appropriate health system budgeting and ensuring continued care provision. Given the scarcity of healthcare personnel, the remote nature of PHCs, and the affordability of WHO-PEN, we conducted a time-and-motion analysis (TMS) to estimate the impact of these interventions on healthcare worker (HCW) time requirements. As these interventions focus on actual care delivery, we anticipate changes in care delivery for follow-up patients, as new patients will still undergo screening and initiation. The findings from the TMS analysis, combined with cost data, facilitated a comprehensive bottom-up costing assessment, providing estimates of the costs associated with delivering diabetes and hypertension care at PHCs from the provider’s perspective in low-resource settings.

## Methods

Detailed description of the study objectives, setting, design, and participant enrolment can be found in the protocol paper [15].

### Time-and-motion analysis

#### Data collection

Data collection took place at 28 randomly selected PHCs across Eswatini’s four regions. Each clinic was visited twice during a 17-day period in June–July 2022, except for one clinic where permission was denied. A team of four collectors covered four different PHCs each day. Collectors performed direct and continuous observations of tasks using timekeeping devices to record activities throughout the workday.

Amongst the nursing cadre observed at PHCs, the ‘expert client’ is a specialized cadre of healthcare personnel, which were initially recruited to cater to patients affected by the human immunodeficiency virus (HIV), as well as those with a DM-HTN comorbidity. Observed nursing cadres include general nurses, nursing sisters, and nurse assistants.

A paper-based Time Management System (TMS) data collection tool was used to monitor healthcare workers’ (HCWs) time utilization across selected facilities, segmented into three sections: healthcare personnel data (A), facility characteristics (B), and the HCW time-tracking tool (C). The tool identified 19 activities related to patient care, administration/meetings, and idle time/breaks, noting that breaks and idle times—spanning lunch, coffee breaks, downtime from low patient volume, and facility cleaning—are essential for productivity and influenced by factors like patient flow. Section C specifically documented the activities of all employee cadres directly involved in patient care, grouping all care activities per patient for data entry.

#### Estimating the total number of patient visits

Our data collection primarily focused on characterizing the composition of care including categorizing new patient visits, DM/HTN patient care, non-DM/non-HTN related patient care, and observed patient visit numbers during the hours the data collectors were present. The TMS data-collection tool and further details on collection procedure and estimation of total patients are provided in Supplement A and B (Additional files 1 and 2).

### Bottom-up costing

We conducted bottom-up costing exercise to assess the financial implications of scaling-up the WHO-PEN interventions for diabetes and hypertension care in Eswatini on a national scale. This analysis was conducted separately for clinics implementing the interventions and those following standard-of-care protocols.

Using demographic, programmatic, financial, and incidence data, we conducted a comprehensive cost assessment for the calendar year 2022–2023. The individual cost estimates derived from the bottom-up costing approach were aggregated to yield comprehensive summary totals. This detailed breakdown includes costs associated with the use of various diagnostic devices for diabetes and hypertension care on a per-patient basis. In addition, it takes into account the need for oral medication and quantifies the time spent by healthcare professionals in providing these interventions. Costs were converted from Eswatini Lilangeni (SZL) to United States Dollar (USD) using the exchange rate from June 2023 ($1 = SZL 18.867) [7].

The aim of this study was to calculate the cost of providing NCD care for diabetes and hypertension in PHCs (excluding the rural health motivators and Public Health Units) in Eswatini from the government’s perspective with the WHO-PEN@Scale interventions. Therefore, we only look at care provision in publicly run PHCs and not private clinics.

Our cost estimates rely on patient volume data derived from the TMS model. Our bottom-up costing analysis is tailored to the number of new-patient DM/HTN visits and follow-up DM/HTN patient visits observed at the facilities in the TMS study. This distinction enhances the precision of our estimates, as new-patient visits are more time-consuming and expensive. Additionally, we conducted a probabilistic uncertainty analysis to establish an expected range for the average DM/HTN patient capacity across PHCs in Eswatini. Further information on data sources, medications, diagnostics tools, sensitivity analysis, and costing procedures are provided in supplement C.

#### Data analysis

The analysis involved calculating HCW time per patient, patient volume, workday duration, average break time, and the proportion of time across different activities. We considered both time of day (morning and afternoon) and patient type (new vs. follow-up) in this analysis. To assess differences in means for critical variables between the two arms, we employed an ANOVA test. All analyses were conducted using STATA (version 16) and Microsoft Excel.

## Results

### Sample characteristics

Overall, 141 different HCWs were monitored providing care to 1171 patients over 17 days of observation for a total of 279 h of HCW shadowing. Of the 1171 patients seen, 353 were DM/HTN or DM/HTN comorbid patients and 818 were non-NCD patients. The share of patients with HIV and DM/HTN comorbidities was less than 2% (23). 864 patients visited the PHCs and were observed in the morning hours before 12:00 while the rest 307 were observed in the afternoon.

#### Primary healthcare clinics

Table 1 gives an overview of the characteristics of the PHCs included in the TMS analysis. A total of 28 facilities were visited twice for combined data on 56 visits, apart from Mgazini Nazarene clinic which was visited thrice and Lavumisa clinic which was visited only once. These facilities were evenly distributed across Eswatini’s four regions, with an equal split between control and intervention groups. 327 employees were observed across these facilities, averaging 12 per facility. Specifically, there were 105 nurses across various cadres, averaging four per PHC. The distribution of employee cadres was consistent across intervention arms, regions, and clinic volumes, as confirmed by a one-way ANOVA test.

**Table 1:** Characteristics of facilities included in the TMS data collection

Item	Overall	SOC	Intervention
Arm type, *n *(% of total)	28	14 (50%)	14 (50%)
Volume^a^			
Low volume, *n* (%)	20	10 (36%)	10 (36%)
High volume, *n* (%)	8	4 (14%)	4 (14%)
Region			
Hhohho, *n* (%)	7	3 (11%)	4 (14%)
Lubombo, *n* (%)	9	5 (18%)	4 (14%)
Shishelweni, *n* (%)	5	3 (11%)	2 (7%)
Manzini, *n* (%)	7	3 (11%)	4 (14%)
Employees (present on the day of visit)			
All staff, *n* (mean, %)	327 (12)	181 (13, 55%)	146 (10, 45%)
All nurses, *n* (mean, %)	105 (4)	57 (4, 54%)	48 (3, 46%)

^a^Low-volume clinics are those with less than 100 patients a day

### Human resource needs

Table 2 presents the reported workday duration of HCWs and observed idle time. We observed a statistically significant difference in the reported workday duration between HCWs in the control and intervention-arm clinic, with the latter group spending, on average, 19 min more at a PHC. This difference was consistent among all nursing staff and expert client employees in intervention-arm clinics, with each spending on average, 27 and 26 more minutes, respectively, as compared to their counterparts in the control-arm clinics.

**Table 2:** Duration of average workday and idle time

Result	Overall	SOC	Intervention
Duration of average reported workday (in HH:MM)			
All staff, mean (95% CI)	8 h 27 m (8 h 22 m–8 h 32 m)	8 h 17 m (8 h 09 m–8 h 24 m)^***^	8 h 36 m (8 h 29 m–8 h 42 m)^***^
Nursing staff	8 h 25 m (8 h 18 m–8 h 31 m)	8 h 10 m (7 h 56 m–8 h 24 m)^***^	8 h 37 m (8 h 32 m–8 h 41 m)^***^
Expert client	8 h 26 m (8 h 15 m–8 h 36 m)	8 h 11 m (7 h 51 m–8 h 32 m)^***^	8 h 37 m (8 h 30 m–8 h 44 m)^***^
All staff except nursing staff & expert client	8 h 28 m (8 h 21 m–8 h 36 m)	8 h 22 m (8 h 12 m–8 h 32 m)	8 h 35 m (8 h 23 m–8 h 47 m)
Idle time, including breaks			
Duration of total idle time in minutes, mean (95% CI)	62 (53–71)	69 (57–81)	57 (44–70)
Average idle time duration per HCW, mins	33 (30–36)	37 (32–41)^**^	30 (26–34)^**^
Average idle time duration per HCW < 12:00, mins	23 (19–27)	24 (18–30)	23 (18–28)
Average idle time duration per HCW > 12:00, mins	42 (38–47)	45 (40–51)	40 (33–46)

^**^p < 0.005, ^***^p < 0.001, () 95% CI

Idle time observations revealed no significant difference in total duration between the two implementation arms. Additionally, the average idle-time duration was 7 min shorter at facilities offering the WHO-PEN interventions. Idle time increased significantly in the afternoon, with HCWs spending 19 more minutes per idle time session after 12:00 compared to morning hours.

The time spent by HCWs per patient stratified by patient type and type of visit are presented in Table 3. HCWs in PHCs implementing the WHO-PEN interventions spent 2 min less per patient seen on average as compared to those in the control-arm clinics. Stratifying the impact of the interventions by patient visit type, we can see that HCWs need on average 1.8 min more to cater to patients visiting the clinic for the first time as compared to those coming in for a follow-up visit. This effect is more pronounced for DM/HTN patients (5.5 min) as they need to undergo screening and counselling before they can be enrolled and provided with care at the facility. The trend in increased average workday duration as seen in Table 1 is reflected in the average number of all patients seen (24 and 18 in the intervention and control-arm, respectively).

**Table 3:** Time per patient and number of observed patients

Result	Overall	SOC	Intervention
Time per patient (mean, 95% CI)			
Time per patient, mins	8.9 (8.6–9.2)	9.9 (9.4–10.4)^***^	8.1 (7.8–8.5)^***^
Time per DM/HTN patient, mins	9.6 (9.1–10.2)	10.9 (9.9–11.8)^***^	8.8 (8.2–9.3)^***^
Time per non-DM/non-HTN patient, mins	8.8 (8.4–9.2)	9.6 (9.0–10.2)^***^	8.2 (7.8–8.7)^***^
Time per patient by visit type (mean, 95% CI)			
Time per new patient visit	10.4 (9.9–11.2)	11.0 (9.9–12.1)^*^	9.6 (8.6–10.6)^*^
Time per follow-up patient visit	8.6 (8.2–8.9)	9.6 (9.0–10.2)^***^	7.9 (7.6–8.3)^***^
Time per DM/HTN new patient visit	15.0 (11.0–19.1)	16.7 (11.1–22.2)	11.0 (7.5–14.4)
Time per DM/HTN follow-up patient visit	9.3 (8.8–9.8)	10.2 (9.3–11.0)^***^	8.7 (8.1–9.3)^***^
Number of patients observed per HCW^a^			
Average number of all patients seen	8 (7–9)	8 (7–9)	8 (6–9)
Average number of DM/HTN patients seen	2 (2–3)	2 (2–3)	2 (2–3)
Average number of non-DM/non-HTN patients seen	6 (5–6)	6 (5–7)	5 (4–7)
Number of patients observed (by facility, daily)			
Average number of all patients seen	21 (18–24)	18 (14–22)^***^	24 (20–29)^***^
Average number of DM/HTN patients seen	6 (4–9)	5 (1–9)^***^	7 (4–10)^***^
Average number of non-DM/non-HTN patients seen	15 (12–17)	13 (10–15)^***^	17 (13–21)^***^

^*^p < 0.01, ^***^p < 0.001

^a^Patients observed per HCW in observed time; doesn’t represent all patient visits for the PHC

Table 4 shows the proportion of observed time spent by HCWs on different activities. On average, a HCW spends 71% of their time attending to patients and on provision of care. This was higher in intervention clinics where HCWs spent close to 73% of their time on patient care as compared to 69% in control-arm clinics. A third of the time of a HCW was spent, on average, on idle time.

**Table 4:** Proportion of HCW time spent on different activities

Result	Overall	SOC	Intervention
Proportion of observed time spent on^a^ (%)			
Patient care	71.08	68.61^***^	72.82^***^
Meetings	7.03	7.03	–
Idle time without patients (incl. breaks)	33.80	35.29	32.61

^***^p < 0.001

^a^Proportions presented are averaged over proportions of individual HCWs and thus do not add up to 100

#### Estimated number of total patient visits

**Fig. 1:**
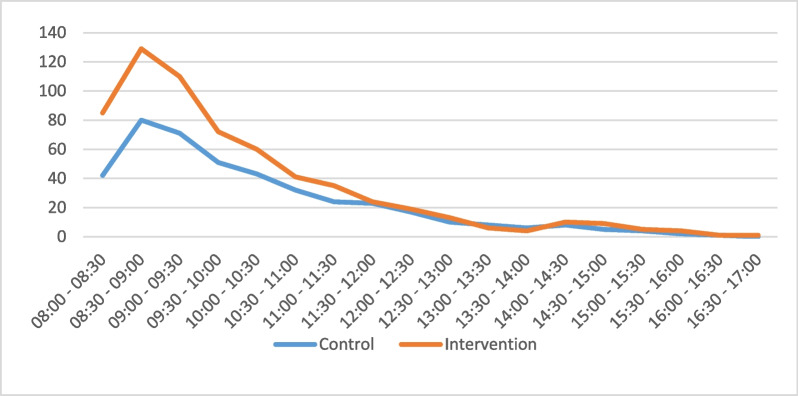
Modelled patient visits by arm at TMS facilities

We estimated the total anticipated number of DM/HTN patient visits at TMS facilities for both the control and implementation arms over a 2-day period. Specifically, we projected 1098 patient visits for the control-arm TMS facilities and 1445 for the intervention-arm facilities. Modelled patient visit numbers at the TMS facilities can be seen in Fig. 1.

### Human resource costs

With a 61% combined prevalence rate, Eswatini has an estimated 123,472 adult cases (aged 40 and above) of diabetes and hypertension, including comorbidities. Out of these, 14,736 are estimated to have diabetes (10%), 122,133 to have hypertension (90%), and 13,397 to suffer from comorbid conditions (10%). According to 2022 CMIS data, there were 145,764 visits for diabetes/hypertension care at PHCs, by 34,803 unique patients, averaging 6.52 visits per patient. This indicates that only 25% of the estimated patient population sought care in 2022. Projecting a full implementation of the WHO-PEN initiative, assuming an average of 6.52 annual visits per estimated patient, could result in 805,039 DM/HTN patient visits at PHCs yearly, marking a 5.5-fold increase from 2022’s patient volume, or realizing only 18% of potential patient visits. Table 5 provides detailed demographics for this population in Eswatini.

**Table 5:** Population under consideration in Eswatini

Population	Estimate (95% CI)
Total population, 40 years and above	223,277
Estimated diabetes patients	14,736 (12,504–16,969)
Estimated hypertension patients	122,133 (119,990–124,365)
Estimated comorbid patients	13,397 (11,164–15,629)
Total estimated DM/HTN patients	123,472 (121,239–125,705)
Total estimated DM/HTN patient visits in 1 year	805,039 (790,481–819,596)

From the TMS analysis, we know that new-patient visits for DM/HTN care are more time-consuming compared to follow-up patient visits, and WHO-PEN interventions streamline care delivery, requiring less time than the control-arm approach. In a scenario of universal scale-up, we estimate that providing DM/HTN care to all estimated patients would require at least 558 nurses with the WHO-PEN interventions, and 981 nurses with standard-of-care. These numbers exclude comprehensive care for non-diabetes and non-hypertension patients, which would necessitate more resources. Considering economic opportunity cost of training the required nurses, the personnel costs of a universal scale-up amount to $4.5 million for the intervention arm and $8.05 million for the control arm, respectively.

The annual costs for supplying diabetes and hypertension medications per estimated patient were calculated $7.71 and $3.15 (Additional file 3: Supplement C Table S4). Given the larger burden of hypertensive patients, the total costs of providing hypertension drugs to all patients was estimated at $384,780. For all diabetic patients, the total annual drug costs were estimated to be $113,420.

**Fig. 2:**
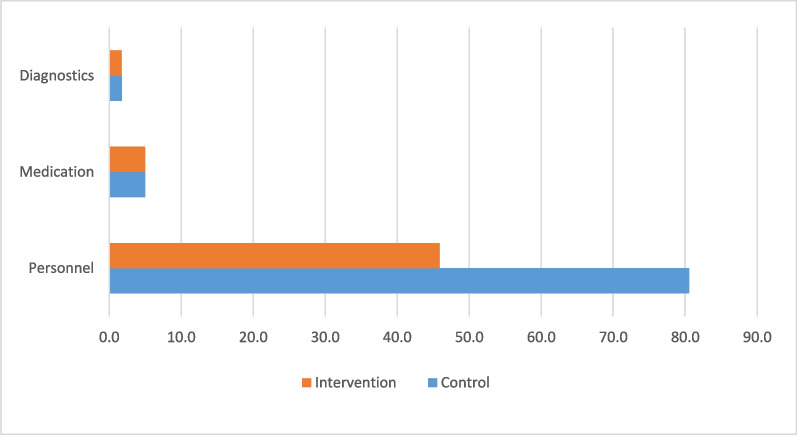
Annual cost of scale-up of WHO-PEN interventions in Eswatini (million USD)

The overall costs for diagnostic equipment varied between the control and intervention arms, contingent upon the number of facilities needed to accommodate all projected patients. For a national-level scale-up, the annual diagnostic costs for a sphygmomanometer, a glucometer, and glucose test strips would amount to $173,310 and $174,900 for the intervention- and control-arm, respectively. The decomposition of these costs is graphed in Fig. 2.

Table 6 presents an overview of the cost of scaling up WHO-PEN interventions in Eswatini under a high-prevalence scenario. The total annual cost of scaling up WHO-PEN interventions to all estimated DM/HTN patients in Eswatini is $5.25 million, equivalent to 6% of the 2022–23 budget of the Eswatini Ministry of Health [4]. Expanding standard-of-care coverage would cost 66% more at $8.73 million. The cost per DM/HTN patient visit amounts to $6.53 with a scale-up and $10.85 in the control-arm scale-up. Additional details on cost of scale-up with different approaches under alternative disease-prevalence scenarios are provided in the Appendix (Additional file 3: Supplement C).

**Table 6:** Costs of scale-up of WHO-PEN in Eswatini (in millions)

Cost (in USD)	SOC (95% CI)	Intervention (95% CI)
Personnel	8.05 (6.44–10.46)	4.58 (3.68–5.82)
Salary costs	7.92 (6.38–10.28)	4.51 (3.62–5.72)
Training costs	0.13 (0.10–0.17)	0.07 (0.06–0.09)
Medication	0.498 (0.471–0.519)	0.498 (0.471–0.519)
Diagnostics	0.175 (0.172–0.179)	0.173 (0.168–0.174)
Total cost	8.73 (7.11–11.13)	5.25 (4.34–6.49)

## Discussion

This study offers a comprehensive analysis of the human and financial resources required for implementing WHO-PEN interventions in resource-constrained settings. Through a comparative evaluation with standard-of-care approaches, we explore the feasibility of expanding care provision, quantify the human resource needs, and present an in-depth breakdown of HCW time requirements both within and beyond the WHO-PEN framework.

### Summary of findings

Our results suggest that there is an increased burden on HCWs of the WHO-PEN interventions in implementing clinics as compared to the standard-of-care arm, especially on nurses and expert clients who are significantly impacted. The results also indicate that these two cadres of care bear the brunt of the intervention, underlining the important role of the expert clients in supporting the implementation of the interventions. Average total break duration does not differ between the two arms of the study, but the difference in the duration of a single break shows that HCWs in intervention clinics were taking shorter but more frequent breaks, further underlying the increased workload of HCWs.

PHCs with WHO-PEN interventions saw more patients but spent less time per patient, suggesting the interventions improve care efficiency, particularly with the fast-track differentiated service delivery (DSD) model for quicker medication access. This efficiency benefit appears to extend to non-DM/non-HTN patients as well, indicating broader impacts of the interventions that warrant further investigation.

Observing patient visits at the HCW and facility levels revealed several key findings. At the HCW level, we did not observe any significant differences in the number of patient visits. This might be attributed to the lower total observation time in intervention-arm clinics (Additional file 2: Supplement B Table S1). At the facility level, we observed a significant difference in the number of patients seen between intervention- (*n* = 24) and control-arm facilities (*n* = 18). The trend of a ‘morning peak’ in patient volume was evident at this level, with an average of 13 patients seen in the morning and four in the afternoon at PHCs (Additional file 3: Supplement C Table S6). This trend held for both DM/HTN and non-DM/non-HTN patients, though it was more pronounced for DM/HTN patients. Furthermore, HCWs also experienced lower idle time and took shorter breaks during the morning hours, reflecting this morning peak in patient visits.

The observed “morning peak” in patient volume at healthcare facilities, particularly among diabetes (DM) and hypertension (HTN) patients, can be attributed to several key factors. First, a large majority (94%) of these patients are returning for follow-up visits, with DM patients often advised to fast before screenings, prompting them to visit early. Additionally, the lack of fixed appointment times leads patients to arrive early to minimize waiting. Other factors, such as long travel distances and the desire to have the rest of the day free, also contribute to early arrivals.

We also observe shorter break durations and a faster processing time of patients in the morning hours for non-DM and non-HTN patients in the DSD arm, potentially indicating the task-shifting HCWs might be undertaking to deal with the larger volume of DM and HTN patients (Additional file 3: Supplement C Table S6). Similarly, intervention-arm clinics experience shorter break durations and faster patient-processing times compared to control-arm clinics, indicating that the maintenance of care quality comes at the cost of HCWs’ idle time. Without an influx of more HCWs, this performance would be difficult to sustain over time. A variety of possibilities to this effect has been explored previously in the SSA context [8].

Our cost estimates focus specifically on the resources allocated to diabetes and hypertension care. The results indicate that scaling-up diabetes and hypertension care to all estimated patients in Eswatini is cheaper with the WHO-PEN interventions as compared to the standard-of-care. Personnel costs represent the most substantial portion of both scenarios’ overall costs. However, there is a notable disparity in personnel costs under the intervention arm, primarily due to the higher patient volume seen at the intervention-arm facilities.

### Findings in context

We expected to see no significant impact of the intervention on the time spent per new DM/HTN patient as they need to undergo screening, testing, diagnosis, and enrolment before they can be provided care. None of these activities were affected by the WHO-PEN interventions. Our results conform to our hypothesis as well as with similar literature. Other TMS studies in Eswatini have quantified the impact of screening for diabetes and hypertension in Eswatini, which leads to a significant rise in time requirement from 4 to 12 min per patient [12]. Our findings complement those from other countries in similar settings as well, in terms of the proportion of time spent on different activities of care and idle time [9].

A literature review on the costs of treating hypertension in 11 SSA countries found a large variation in the treatment costs from both patient and provider perspective across countries, underlining the difficulty in generalizing such data [5]. Additionally, only 25% of all SSA countries reported such data, further highlighting the need to quantify human resource costs for provision of care in such settings. Costs of regular outpatient treatment for hypertensive patients (from a provider’s perspective) ranged from under $10 in Ghana and Kenya to over $15 in Guinea and Rwanda. The same study found costs of treatment for outpatient visits at PHCs to be significantly lower than both outpatient visits at hospitals and inpatient visit costs. Another study in the South African context quantifying the costs for scaling up treatment for diabetes and hypertension according to the WHO-PEN guidelines found the annual per patient cost for treating diabetes and hypertension to be $134 and $27, respectively [2]. However, these were calculated as the lifetime costs for each patient borne by the provider and included insulin treatment as a cost.

### Practical implications

Our findings on the human resource needs and costs for expanding care under the WHO-PEN interventions can guide policy to provide efficient care for DM and HTN patients in Eswatini and countries with comparable care settings. Data on workday duration of a HCW and the time required for administering care to patients by type of visit can be used to optimize workflow, as well as to effectively plan any future programmes involving screening for cardiovascular diseases.

Policies aimed at smoothening and redistributing the patient load from the morning to the afternoon hours might free-up HCW time in the morning hours and further boost efficiency. Since DM/HTN patients (especially diabetes patients) cannot arrive later in the day due to constraints on their eating times, focusing on DM/HTN patients in the morning by having special DM/HTN-only hours might help decongest demand for healthcare by patients seeking non-diabetes care. Similarly, having non-DM/non-HTN hours in the afternoons where they are prioritized might generate the same effect with the addition of boosting healthcare demand in the afternoons.

By strategically implementing such policies, healthcare facilities can aim to optimize HCW productivity, streamline patient flow, and enhance overall healthcare service delivery, ultimately contributing to improved outcomes for DM and HTN patients and the broader community.

### Strengths and limitations

Our study is the first to present a detailed description of the human resource needs for providing and expanding care to DM/HTN patients in Eswatini under a standard-of-care scenario and under the WHO-PEN@Scale interventions. Our data collection, which randomly selected 28 PHCs while accounting for the treatment arm, patient volume, and geographic location, ensures our data to be representative for a small country such as Eswatini.

We also addressed the possibility of a Hawthorne Effect (HE) among HCWs during observation. HCWs were informed beforehand that data collection was for research purposes only, not performance evaluation. Previous research suggests that factors like observation duration, task complexity, and workload influence HE potential [10]. Given the high patient volume, relatively simple tasks observed, and multiple observation sessions over two days, we believe any HE effect was minimal. Additionally, since HCWs from both study arms were observed, any differences observed are likely due to the intervention rather than HE.

There are certain limitations to consider regarding the TMS data and our analysis. Firstly, our data collection does not directly incorporate non-nursing staff contributions to patient care, potentially underestimating the actual time needed for care provision. Additionally, we do not account for the varied implementation strategies of WHO-PEN in our analysis, which could impact outcomes. Moreover, our modelling assumptions, such as consistent patient behavior and visit patterns, may not fully capture real-world complexities, necessitating further research to refine these parameters.

Further information on individual, detailed tasks of providing DM/HTN care such as drawing blood glucose, obtaining vital signs and blood pressure, providing consultation, etc., are needed to identify opportunities for improving care provision at the individual nurse level. Assistance and contribution of non-nursing cadres in providing DM/HTN care can be ascertained by expanding the TMS data collection tool to include their support. Estimating the time required to achieve a complete scale-up of the coverage of our interventions is currently outside the scope of our study, as this is influenced by a variety of factors ranging from infrastructural challenges to cultural practices.

Our study offers valuable insights for SSA countries with low-resource settings and healthcare workforce shortages, especially those operating in rural areas. However, we must acknowledge limitations in generalizability due to the experimental nature of our study and the dynamic health system landscape, including challenges from COVID-19, which affected the Eswatini government's capacity to fully integrate WHO-PEN interventions is affected. These factors call for caution when applying our findings beyond our specific context.

## Conclusion

We assessed the impact of implementing WHO-PEN interventions for diabetes and hypertension care in PHCs in Eswatini on HCWs’ time. Our findings reveal increased efficiency in delivering care for these diseases, accompanied by an increased workload for HCWs, with no adverse effects on non-DM/non-HTN care. Without an increase in the healthcare workforce in the country, the positive performance of the interventions might not be sustainable in the long-term, thus highlighting the issues of challenges in the creation and retention of healthcare professionals Eswatini and in SSA. While the national-level scale implementation of these interventions holds high potential for enhancing healthcare access in the country, further research is needed to understand how the three DSD arms impact HCW time and to identify the best-performing arm.

The WHO-PEN interventions for diabetes and hypertension control in Eswatini have the potential to alleviate the disease burden at tertiary healthcare facilities in the country. The implementation of WHO-PEN interventions in Eswatini could enable the primary healthcare system to accommodate more patients at a lower cost. Nevertheless, the decision to scale-up these interventions should also consider factors such as their impact on population health, health equity, and the cost-effectiveness of the different intervention arms.

### Supplementary Information

Supplementary Material 1. Supplement A: TMS data collection tool.

Supplementary Material 2. Supplement B: Additional details for time-and-motion analysis.

Supplementary Material 3. Supplement C: Additional details for bottom-up costing.

## Data Availability

The deidentified full dataset from our household survey for the overall WHO-PEN@Scale in Eswatini, along with all code for data management and analysis will be posted in a publicly accessible data repository upon publication of the findings for the primary endpoints.  The specific dataset analysed during the current study are available from the corresponding author on reasonable request.

## Abbreviations

ANOVA: Analysis of variance
CI: Confidence interval
CHAI: Clinton Health Access Initiative
COVID-19: Coronavirus disease 2019
CMIS: Client management information system
DM: Diabetes mellitus
DSD: Differentiated service delivery
GBD: Global burden of disease
HCW: Healthcare worker
HE: Hawthorne effect
HIV: Human immunodeficiency viruses
HTN: Hypertension
NCD: Non-communicable diseases
PHC: Primary healthcare clinic
SOC: Standard of care
SSA: Sub-Saharan Africa
SZL: Eswatini Lilangeni
TMS: Time-and-motion study
USD: United States Dollar
WHO-PEN: World Health Organization’s Package of Essential Non-Communicable Disease (NCD) Interventions
