# Supplementary material for: Human and financial resource needs for universal access to WHO-PEN interventions for diabetes and hypertension care in Eswatini: results from a time-and-motion and bottom-up costing study
Source: Hum Resour Health. 2024 May 27;22:32. doi: 10.1186/s12960-024-00913-0 (PMC11131333; doi:10.1186/s12960-024-00913-0)
Supplement: Supplementary file 1 — Supplementary Material 1. Supplement A: TMS data collection tool. [file 12960_2024_913_MOESM1_ESM.docx]

**Title**: Human and financial resource needs for universal access to WHO-PEN interventions for diabetes and hypertension care in Eswatini: Results from a time-and-motion and bottom-up costing study

**Authors**: Harsh Vivek Harkare, Brianna Osetinsky, Ntombifuthi Ginindza, Bongekile Thobekile Cindzi, Nomfundo Mncina, Babatunde Akomolafe, Lisa-Rufaro Marowa, Nyasatu Ntshalintshali, Fabrizio Tediosi

# Additional file 1

# Supplement A: TMS data collection tool

## Section A

**A01. Date**: | | |/| | |/| | | | |

Day /Month/Year

**A02. Name of interviewer**:

**A03. Time Enumerator arrived at health facility day 1 (hh:mm)**

**A04. Time Enumerator Departed facility (hh:mm)**

**A05 Code of health facility**:

**A06** Total number of health workers present, by cadre, at the facility on the data collection day and, if absent, reason for absence. If there are no workers of a particular cadre enter 0 for both present and absent. For Other, use each line for different Cadres of other employees.

| **Worker cadre:** | **Number Present:** | **Number absent:** | **Reason for absence:** | **Number serving NCD patients today** | **Time Arrive (reported or observed)** | **Time Depart (reported or observed)** |
| --- | --- | --- | --- | --- | --- | --- |
| **A) Nurse** |  |  |  |  |  |  |
| **B) Nurse Sister** |  |  |  |  |  |  |
| **C) Nurse assistant** |  |  |  |  |  |  |
| **D) Expert client** |  |  |  |  |  |  |
| **E) Phlebotomist** |  |  |  |  |  |  |
| **F) Pharmacist** |  |  |  |  |  |  |
| G) **Lay Cadre**  **(works with patients)** |  |  |  |  |  |  |
| **H) Other, specify here:** |  |  |  |  |  |  |
| **I) Other, specify here: :** |  |  |  |  |  |  |
| **J) Other, specify here: :** |  |  |  |  |  |  |
| **K) Other, specify here: :** |  |  |  |  |  |  |
| **L) Other, specify here: :** |  |  |  |  |  |  |

|  |  | | **Codes for absence reasons:** | |
| --- | --- | --- | --- | --- |
|  | 1= | | Unknown | |
|  | 2= | | Training/education | |
|  | 3= | | Home visits | |
|  | 4= | | Attend to a medical emergency | |
|  | 5= | | Clinical work at another facility | |
|  | 6= | | Sick leave | |
| 7= | | Maternity leave | |  |
| 8= | | Leave for holidays, funerals, etc. | |  |
| 9= | | Other, specify in table | |  |

**A07** Notes for anything to do with B02 including if some workers come at a different time from the others in their Cadre or any other information about arrival/departures/absences and serving NCD patients

## Section B

Now we will ask about the NCD care delivery options available at this clinic (Only to be answered once)

| **B01** | Is there a specific NCD care day? | - 1 Yes - 0 No |
| --- | --- | --- |
| **B02** | How often does an NCD specific Care Day Occur | - 1 Daily - 2 Weekly - 3 Monthly - 99 Not Applicable |
| **B03** | Does this facility offer support for Community Based DSD Clubs? | - 1 Yes - 0 No |
| **B04** | Does this facility offer support for Facility Based DSD Clubs? | - 1 Yes - 0 No |
| **B05** | Does the facility provide the Fast Track option? | - 1 Yes - 0 No |
| **B06** | Who prepares medicines for the DSD groups or fast track?  (Please use code A – L from section B02. If no groups or fast track is available  please enter N/A) |  |
| **B07** | When does the person prepare the medicines for DSD groups or fast track pickup?  (If no groups or fast track is available please enter N/A) |  |
| **B08** | How much time does the preparation of medicines take? Estimate in minutes. (If no groups or fast track is available please enter N/A) |  |
| **B09** | Do stockouts impact the use of NCD care? | - 1 Yes - 0 No |

| **B09a** | If yes (to B09), please explain. (Especially ask about DSD clubs or fast track if they are offered at facility) |
| --- | --- |
| **B10** | Please share other information here that can improve the understanding of how your care is delivered. This can include care protocols including who gets screened for NCDs, or relationships between this and other facilities to mitigate the effect of stockouts. |

## Section C

**C01 Health Worker and time observed:**

| HCW Internal ID  number | HCW gender | Time Begin tracking | Time End Tracking |
| --- | --- | --- | --- |
|  |  |  |  |
|  |  |  |  |
|  |  |  |  |
|  |  |  |  |
|  |  |  |  |
|  |  |  |  |

| **#** | **Nurse Code** | **Activity Code** | **Time Started** | **Time Ended** | **No. of interruptions** | **Non- NCD**  **Care** | **NCD**  **Care** | **NP** | **C** | **Med** | **Ref** | **Notes (Including # if patient leaves and returns so they are linked)** |
| --- | --- | --- | --- | --- | --- | --- | --- | --- | --- | --- | --- | --- |
| 1 |  |  |  |  |  | □ | □ | □ | □ | □ | □ |  |
| 2 |  |  |  |  |  | □ | □ | □ | □ | □ | □ |  |
| 3 |  |  |  |  |  | □ | □ | □ | □ | □ | □ |  |
| 4 |  |  |  |  |  | □ | □ | □ | □ | □ | □ |  |
| 5 |  |  |  |  |  | □ | □ | □ | □ | □ | □ |  |
| 6 |  |  |  |  |  | □ | □ | □ | □ | □ | □ |  |
| 7 |  |  |  |  |  | □ | □ | □ | □ | □ | □ |  |
| 8 |  |  |  |  |  | □ | □ | □ | □ | □ | □ |  |
| 9 |  |  |  |  |  | □ | □ | □ | □ | □ | □ |  |
| 10 |  |  |  |  |  | □ | □ | □ | □ | □ | □ |  |
| 11 |  |  |  |  |  | □ | □ | □ | □ | □ | □ |  |
| 12 |  |  |  |  |  | □ | □ | □ | □ | □ | □ |  |
| 13 |  |  |  |  |  | □ | □ | □ | □ | □ | □ |  |
| 14 |  |  |  |  |  | □ | □ | □ | □ | □ | □ |  |
| 15 |  |  |  |  |  | □ | □ | □ | □ | □ | □ |  |
| 16 |  |  |  |  |  | □ | □ | □ | □ | □ | □ |  |
| 17 |  |  |  |  |  | □ | □ | □ | □ | □ | □ |  |
| 18 |  |  |  |  |  | □ | □ | □ | □ | □ | □ |  |
| 19 |  |  |  |  |  | □ | □ | □ | □ | □ | □ |  |

## Activities recorded in the TMS tool

| **#** | **Activity** | **Category** | **sub-category** |
| --- | --- | --- | --- |
| **1** | **Obtaining vital signs (eg, temp, blood pressure) and/or**  **weighing** | **Patient Care** | **General Care** |
| **2** | **Repeat blood pressure** | **Patient Care** | **NCD care** |
| **3** | **Blood draw glucose check** | **Patient Care** | **NCD Care** |
| **4** | **Hypertension Care** | **Patient Care** | **NCD Care** |
| **5** | **Diabetes Care** | **Patient Care** | **NCD Care** |
| **6** | **Asthma Care** | **Patient Care** | **NCD Care** |
| **7** | **Other NCD Care** | **Patient Care** | **NCD Care** |
| **8** | **HIV care** | **Patient Care** | **Other Care** |
| **9** | **NCD Vitals Check without NCD diagnosis** | **Patient Care** | **NCD Care** |
| **10** | **Consultation** | **Patient Care** | **Other Care** |
| **11** | **In clinic lab** | **Patient Care** | **General Care** |
| **12** | **Filling out patient charts and other patient records** | **Administration** |  |
| **13** | **Other administrative work (specify, if possible)** | **Administration** |  |
| **14** | **Dispensing (Observed)** | **Patient Care** | **General Care** |
| **15** | **Dispensing (Left observer to dispense)** | **Patient Care** | **General Care** |
| **16** | **Meeting** | **Administration** |  |
| **17** | **Lunch break** | **Break** |  |
| **18** | **Coffee/tea break** | **Break** |  |
| **19** | **Other type of break/idle time** | **Break** |  |
