# Supplementary material for: Human and financial resource needs for universal access to WHO-PEN interventions for diabetes and hypertension care in Eswatini: results from a time-and-motion and bottom-up costing study
Source: Hum Resour Health. 2024 May 27;22:32. doi: 10.1186/s12960-024-00913-0 (PMC11131333; doi:10.1186/s12960-024-00913-0)
Supplement: Supplementary file 2 — Supplementary Material 2. Supplement B: Additional details for time-and-motion analysis. [file 12960_2024_913_MOESM2_ESM.docx]

**Title**: Human and financial resource needs for universal access to WHO-PEN interventions for diabetes and hypertension care in Eswatini: Results from a time-and-motion and bottom-up costing study

**Authors**: Harsh Vivek Harkare, Brianna Osetinsky, Ntombifuthi Ginindza, Bongekile Thobekile Cindzi, Nomfundo Mncina, Babatunde Akomolafe, Lisa-Rufaro Marowa, Nyasatu Ntshalintshali, Fabrizio Tediosi

# Additional file 2

# Supplement B: Additional details for time-and-motion analysis

*Data collection*

Data collectors were instructed to shadow each HCW administering care at least once a day for a minimum of 2 hours. If there was only one HCW at the facility, they were observed for the entire observation period. For sections A and B, the head nurse, or if unavailable, a senior nurse was interviewed. Data on other employees such as phlebotomists, pharmacists, lay cadres, etc. was also observed and recorded, if present. The data collectors were instructed to enter the data from their paper-based tool into an Excel workbook. This was uploaded to the data supervisor at regular intervals to check for errors and inconsistencies.

The TMS data collection focused on the medical component of the provision of care including, but not limited to, testing for blood glucose, counselling for care, and dispensing medicines, as well as recording in patient charts and record books while the patients were present, and writing prescriptions. The administrative part of providing care, such as making appointments, conducting triage, calling patients, etc. was outside the scope of this analysis. Resources and time spent on training the nurses for the implementation of the WHO-PEN interventions could not be accounted for in the TMS analysis. Similarly, any supporting tasks conducted by lay cadre or non-nursing cadre to assist the nurses in providing care were also not included.

*Estimating the total number of patient visits*

Our data collection was not aimed at capturing the total patient volume for the entire health centre. Due to the structure of data collection and variable patient volume through the day, in order to model the total DM/HTN patient volume by types of visit, we combined the primary data with routinely collected patient numbers for DM/HTN patients collected through Eswatini’s Client Management information System (CMIS). The CMIS is a digital medical records management system that comprehensively logs all patient visits throughout the country. We utilized patient volume multipliers derived through a probabilistic uncertainty approach; explained in detail in the appendix, to estimate the total patient visits separately for the control and intervention arms, encompassing the facilities included in the TMS analysis.

The CMIS captures essential patient demographics, including age, gender, facility visit, diagnoses, and prescribed treatments. Additionally, during electricity blackouts and maintenance periods affective the system, HCWs complete downtime forms, which are subsequently entered into the CMIS. As a result, the CMIS records effectively mirror the trends in patient visits to primary healthcare clinics in the country.

The time-period of 11:00-11:30 was identified as the time period that matched most-closely with the trend in patient visits across the CMIS and TMS dataset and was thus selected as the referent group for calculating patient visit multipliers. To test the suitability of the referent time period, we conducted a chi-squared goodness-of-fit test to test the fit of the observed and modelled number of patient visits. We developed a probabilistic model to estimate the total number of DM/HTN patient visits based on the observed number of DM/HTN patient visits to control- and intervention-arm PHCs from the CMIS data from the year 2022. A gamma distribution was fitted to the total number of DM/HTN patients seen according to the CMIS data to get a range of corrected estimates of total DM/HTN patient visits. Similarly, the TMS data was also fitted to a gamma distribution with 1000 repetitions to get a range of corrected estimates. Multipliers for patient visits were calculated for 30-min intervals starting from 08:00 until 17:00 with patient visits in the period 11:00- 11:30 as the referent group. The distribution of multipliers for these 30-minute increments were multiplied with the gamma distribution of the observed number of patient visits in control- and intervention-arm clinics as seen in the TMS data to arrive at the expected number of DM/HTN patient visits in half-hour slots. A benefit of calculating separate multipliers for the control- and intervention-arms is that it captures the differential trend in patient visit volume to the intervention-arm clinics, if any. Similarly, a benefit of using a probabilistic uncertainty approach makes our estimates more robust by accounting for the uncertainty in missed patient visits.

To calculate the number of new-patient visits and follow-up patient visits, we used data from the observed TMS data to fit a beta distribution of the share of type of patient visit. This range was applied to the total expected DM/HTN patient visits to get the number of expected new-patient and follow-up patient visits.

Our study estimates the expected number of DM/HTN patient visits from the observed number of patient visits. A reason for the undercounting in our observed data could be due to the design of the TMS data collection tool focusing on the detailed composition of how HCWs spend their time as well as due to the variation in total amount of time spent by data collectors in different PHCs. We correct these biases with the probabilistic uncertainty approach.

***Table S1. Observation times for different HCWs***

|  | **Overall** | **SOC** | **DSD** |
| --- | --- | --- | --- |
| Number of unique HCWs observed over two days | 90 | 37 | 53 |
| Number of HCWs observed across two days | 162 | 66 | 96 |
| Mean observation time, mins | 108 | 124^***^ | 97^***^ |
| Median and IQR of observation time, mins | 67 (60-122) | 119 (64-131) | 65 (60-77) |

***Table S2. Characteristics of studied facilities included in the TMS study***

| Facility name | Nurses | EC | Non-nursing staff | Tot | Region | Volume | Intervention arm |
| --- | --- | --- | --- | --- | --- | --- | --- |
| Acts II Clinic | 5 | 0 | 2 | 7 | Manzini | Low | SOC |
| Bulandzeni Clinic | 3 | 2 | 4 | 9 | Hhohho | Low | SOC |
| Bulunga Nazarene Clinic | 3 | 1 | 2 | 6 | Manzini | Low | SOC |
| Cana Alliance Clinic | 3 | 1 | 2 | 6 | Manzini | High | DSD |
| Endzingeni Nazarene Clinic | 5 | 1 | 1 | 7 | Hhohho | Low | SOC |
| Entfonjeni Clinic | 5 | 2 | 4 | 11 | Hhohho | High | DSD |
| Gilgal Clinic | 6 | 6 | 5 | 17 | Lubombo | Low | SOC |
| JCI Clinic | 3 | 2 | 6 | 11 | Shiselweni | High | SOC |
| Jericho Clinic | 4 | 2 | 6 | 12 | Shiselweni | Low | DSD |
| Lamvelase Zombodze Clinic | 9 | 4 | 2 | 15 | Manzini | Low | DSD |
| Lavumisa Clinic | 3 | 2 | 4 | 9 | Shiselweni | Low | SOC |
| Mafutseni Nazarene Clinic | 5 | 3 | 7 | 15 | Manzini | High | SOC |
| Malindza Refugee Clinic | 6 | 2 | 4 | 12 | Lubombo | High | SOC |
| Mangweni Clinic | 6 | 1 | 9 | 16 | Hhohho | High | DSD |
| Mgazini Nazarene Clinic | 3 | 1 | 1 | 5 | Shiselweni | Low | DSD |
| Mliba Nazarene Clinic | 6 | 4 | 12 | 22 | Manzini | Low | DSD |
| Ndzevane Clinic | 7 | 3 | 8 | 18 | Lubombo | Low | SOC |
| Ngculwini Nazarene Clinic | 4 | 3 | 11 | 18 | Manzini | High | DSD |
| Nkonjwa Clinic | 4 | 1 | 0 | 5 | Lubombo | Low | DSD |
| Peak Nazarene Clinic | 4 | 1 | 1 | 6 | Hhohho | Low | DSD |
| Sigangeni Clinic | 6 | 2 | 2 | 10 | Hhohho | High | SOC |
| Sigcaweni Nazarene Clinic | 4 | 1 | 0 | 5 | Lubombo | Low | SOC |
| Silele Red Cross Clinic | 6 | 2 | 10 | 18 | Shiselweni | Low | SOC |
| Sinceni Clinic | 5 | 2 | 8 | 15 | Lubombo | Low | DSD |
| Siphofaneni Clinic | 12 | 8 | 2 | 22 | Lubombo | High | SOC |
| Tikhuba Clinic | 5 | 6 | 5 | 16 | Lubombo | High | DSD |
| Tsambokhulu Clinic | 7 | 2 | 11 | 20 | Lubombo | Low | DSD |
| Vusweni Clinic | 2 | 1 | 1 | 4 | Hhohho | Low | DSD |

*Goodness-of-fit test for observed vs modelled patients*

A chi-squared goodness-of-fit test was conducted on the observed and modelled number of patient visits at TMS facilities over two days for the time period 11:00-13:00. With a p-value=0.2849, we cannot reject the null hypothesis that the observed and expected values are statistically different.


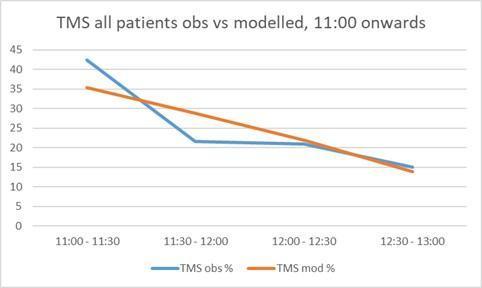
**Figure S1. TMS observed and expected patients visits between 11:00-13:00**

**Figure S2. Observed patients visits by arm and time from CMIS data**


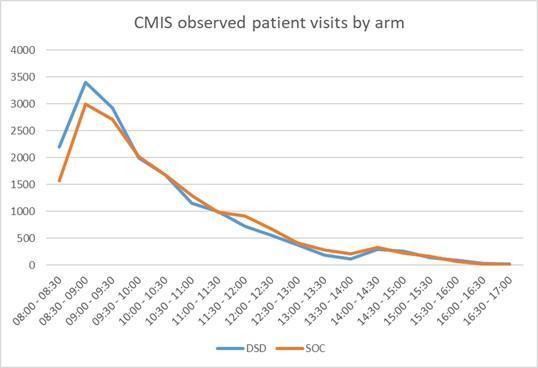


**Table S3. List of disease-specific medication and equipment included**

| Diabetes | Medication | Paracetamol 500mg |
| --- | --- | --- |
|  |  | Metformin 500mg |
|  |  | Metformin 850mg |
|  |  | Glibenclamide 5mg |
|  | Equipment | Glucometer |
|  |  | Glucose test strips |
| Hypertension | Medication | Paracetamol 500mg |
|  |  | Hydrochlorothiazide 25mg |
|  | Equipment | Sphygmomanometer |
