# Supplementary material for: Human and financial resource needs for universal access to WHO-PEN interventions for diabetes and hypertension care in Eswatini: results from a time-and-motion and bottom-up costing study
Source: Hum Resour Health. 2024 May 27;22:32. doi: 10.1186/s12960-024-00913-0 (PMC11131333; doi:10.1186/s12960-024-00913-0)
Supplement: Supplementary file 3 — Supplementary Material 3. Supplement C: Additional details for bottom-up costing. [file 12960_2024_913_MOESM3_ESM.docx]

**Title**: Human and financial resource needs for universal access to WHO-PEN interventions for diabetes and hypertension care in Eswatini: Results from a time-and-motion and bottom-up costing study

**Authors**: Harsh Vivek Harkare, Brianna Osetinsky, Ntombifuthi Ginindza, Bongekile Thobekile Cindzi, Nomfundo Mncina, Babatunde Akomolafe, Lisa-Rufaro Marowa, Nyasatu Ntshalintshali, Fabrizio Tediosi

# Additional file 3

# Supplement C: Additional details for bottom-up costing

*Data sources*

The estimated number of diabetes and hypertension patients in the country were derived from WHO-estimated prevalence for the years 2016 and 2020 respectively. The average annual patient visit figures were sourced from the CMIS. To calculate the overall personnel time requirement for the WHO-PEN scale-up in Eswatini, we used data from the TMS analysis, including the average number of daily DM/HTN visits and the average number of nurses employed at a PHC. Information pertaining to nurse training was obtained directly from the Eswatini Ministry of Health. Finally, the data on personnel salaries, dosage and costs of government-recommended medication, and the number and costs of different diagnostic equipment used in the provision of care was obtained from the Ministry of Health. The treatment guidelines for healthcare provision at the primary healthcare level were aligned with the standards established by the Kingdom of Eswatini, as defined by the Ministry of Health in 2012.

*Bottom-up costing procedure*

The aim of this study was to calculate the cost of providing DM/HTN care for diabetes and hypertension in PHCs from the government’s perspective with the WHO-PEN@Scale interventions. Specifically, we estimate costs of personnel (including general staff training), disease-specific medications, and disease-specific diagnostic equipment. We conduct costing separately for the control arm and the intervention arm under an assumption of 100% coverage of the interventions. Our cost estimates are reflective of a modelled scenario where all estimated diabetes and hypertension patients in Eswatini seek treatment since increasing access is one of the goals of the program. The medicinal- and diagnostic-component of the cost analysis is exclusive to care for diabetes and hypertension patients. Since a HCW rarely provides DM/HTN care exclusively, it is impossible to accurately estimate the time-requirement for DM and HTN care provision only. Thus, the personnel-cost component of the analysis is indicative of the costs required for providing care to all estimated diabetes and hypertension patients in Eswatini along with other NCD and non-NCD patients. The costs do not reflect the cost of training the HCWs for the intervention models specifically. However, economic costs arising from general staff trainings in a given year in Eswatini have been accounted for. We do not account for operating costs of PHCs in terms of their rent and utilities. Initial capital expenditure for setting up new facilities is not captured in our cost estimate.

We assume the cost of training for HCWs to be the average number and duration of training sessions conducted in the country annually. Data on staff training sessions was acquired from the Ministry of Health. The cost estimates do not consider the supplementary costs incurred by HCWs such as travelling to work or the opportunity cost of not going to work. Administrative costs such as supportive supervision and regional coordination are also not included. Costing for DM/HTN-specific diagnostic equipment does not take into account diagnostic tools and general clinical equipment that are used in providing care for various treatments, including for diabetes and hypertension, but not exclusively for them. Examples of such equipment are kidney dishes, examination beds, tables, syringes, gloves, drug bags, bin liners, etc.

Using data from the modelled number of patient visits, we calculated the maximum number of patient visits a single PHC can cater to over one year. This was done separately for new-patient visits and follow-up patient visits. Next, we used data from the WHO’s prevalence estimates of diabetes and hypertension in Eswatini to get the total number of estimated affected people aged 40 and above (WHO, 2016; WHO, 2020). In using the prevalence estimates from the WHO, we choose the higher-end of the specified range of prevalence to derive the maximum possible cost. Combining this with data from the CMIS on average number of annual patient visits, we derived the total number of estimated DM/HTN patient visits over one year. We arrive at the total number of required PHCs and nurses by dividing the estimated number of patient visits by the maximum patient-visit capacity of one facility on average. Personnel costs were calculated by multiplying the salaries of the nursing cadre with the estimated number of nurses. The economic opportunity costs of their time spent in on-the-job training is also considered. Medication costs have been calculated based on the total estimated number of affected patients in the country as well as the recommended mix and dosage of medicines (see Table S1). Diagnostic costs were annualised for the disease-specific diagnostic equipment used exclusively for diabetes or hypertension care. A list of items not included in the costing exercise has been provided above.

In our estimation of the scale-up cost for WHO-PEN in Eswatini, we make several key assumptions:

1. **Patient Visits**: We assume that the number of follow-up patient visits for DM/HTN care to various PHCs remains constant at 6.52 visits per year, as indicated by the CMIS data. Additionally, new patients are expected to visit a PHC an average of 5.52 times within a year after their initial visit.
2. **Patient Volume Trends**: We assume that patient volume trends to different PHCs are uniform across the entire country.
3. **Working Days**: We estimate the effective number of working days in Eswatini to be 220 days per year, accounting for holidays and nurses’ sick leaves.
4. **Depreciation Rate**: To calculate the annualized costs of diagnostic equipment, we assume a depreciation rate of 10% for equipment for which specific information, such as the glucometer, was not available.

To test the robustness of our results, we conducted the costs assessment with a different set of modelled patient visit numbers derived using a simple multiplier approach instead of the probabilistic uncertainty multiplier approach.

**Items excluded from the costing exercise**

- Any capital costs
- Utility costs of PHCs
- Costs of administration and supervision at the regional and national level

**Sensitivity analysis for bottom-up costing**

*Multiplier approach for deriving modelled number of patient visits and cost data*

We estimated the total number of patient visits to the facilities included in the TMS analysis, for the control- and intervention-arm separately using a multiplier approach. The time-period of 11:00-11:30 was identified as the time period that matched most-closely with the trend in patient visits across the CMIS and TMS dataset and was thus selected as the referent group for calculating patient visit multipliers. We then use a simple mathematical model to estimate the total number of patient visits based on the observed number of patient visits to all control- and intervention-arm PHCs from the CMIS data from the year 2022. Multipliers for patient visits were calculated for 30-min intervals starting from 08:00 until 17:00 with patient visits in the period 11:00-11:30 as the referent group. Multipliers for these 30-minute increments were multiplied with the observed number of patient visits in control- and intervention-arm clinics as seen in the TMS data to arrive at the expected number of patient visits in half-hour slots. A benefit of calculating separate multipliers for the control- and intervention-arms is that it captures the differential trend in patient visit volume to the intervention-arm clinics, if any.

| **Table S4. Unit cost of DM/HTN** **medication** | | | | | | | |
| --- | --- | --- | --- | --- | --- | --- | --- |
| **Diabetes** | | | | | | | |
| **Medicines** | **Quantity** | **Proportion** | **Pack of** | **Cost (USD)** | **Description** | **Proportional cost (USD)** | **Annual costs** |
| Paracetamol 500mg | 1 | 100% | 5000 | 38.40 | Pack of 30 tablets inteded for 30 days of use | 0.23 | 2.76 |
| Metformin 500mg | 1 | 50% | 500 | 2.50 | Pack of 30 tablets intended for 30 days | 0.15 | 1.80 |
| Metformin 850mg | 1 | 50% | 500 | 3.52 | Pack of 30 tablets intended for 30 days | 0.21 | 2.53 |
| Glibenclamide 5mg | 1 | 100% | 1000 | 1.70 | Pack of 30 tablets intended for 30 days | 0.05 | 0.61 |
| Total drug cost per diabetes patient visit | | |  |  |  | 0.64 | 7.71 |
| **Hypertension** | | | | | | | |
| Paracetamol 500mg | 1 | 100% | 5000 | 38.40 | Pack of 30 tablets inteded for 30 days of use | 0.23 | 2.76 |
| Hydrochlorothiazide 25mg | 1 | 100% | 1000 | 2.31 | Pack of 14 tablets | 0.03 | 0.38 |
| Total drug cost per hypertension patient visit | | |  |  |  | 0.26 | 3.14 |

To calculate the number of DM/HTN patient visits from the expected all patient visits, we derived the share of DM/HTN patients from the total all patient visits being observed in 30-min slots from 08:00 until 17:00. This was done separately for the control- and intervention-arm clinics to account for any differences in DM/HTN patient volume. Given the lack of data on patient visits between 08:00-08:30, the DM/HTN share of patient visits from 08:30-09:00 was used instead. Similarly for the time period 14:00-14:30 and 14:30-15:00 in control-arm clinics, the share of DM/HTN patients visits from 13:30-14:00 was used.

| **Table S5. Costs of scale-up of WHO-PEN with multiplier approach (in millions)** | | |
| --- | --- | --- |
| Costs (in USD) | SOC scale-up | DSD scale-up |
| Personnel salary costs | 7.72 | 4.94 |
| *Personnel training costs* | 0.12 | 0.08 |
| Drug costs | 0.49 | 0.49 |
| Diagnostic equipment costs | 0.16 | 0.15 |
| Total cost of care | 8.52 | 5.68 |

| **Table S6. Time per patient and number of observed patients by time of day** | | | | |
| --- | --- | --- | --- | --- |
| Result | *By arm and time of day* | | | |
|  | SOC <12:00 | DSD <12:00 | SOC >12:00 | DSD >12:00 |
| Time per patient, mean 95% CI |  |  |  |  |
| Time per patient, minutes | 9.8 (9.2-10.4)^***^ | 7.8 (7.4-8.2)^***^ | 10.3 (9.3-11.3)^***^ | 9.0 (8.4-9.7)^***^ |
| Time per DM/HTN patient, minutes | 11.0 (9.8-12.1)^***^ | 8.8 (8.1-9.5)^***^ | 10.6 (9.0-12.2)^***^ | 8.6 (7.8-9.4)^***^ |
| Time per non-DM/non-HTN patient, minutes | 9.4 (8.7-10.1)^***^ | 7.8 (7.2-8.3)^***^ | 10.0 (8.6-11.5) | 9.2 (8.3-10.1) |
| Time per patient by visit type, mean 95% CI |  |  |  |  |
| Time per new patient visit | 11.1 (9.8-12.4)^***^ | 9.1 (8.2-10.1)^***^ | 10.5 (8.3-12.7) | 13.1 (7.3-18.9) |
| Time per follow-up patient visit | 9.4 (8.7-10.0)^***^ | 7.6 (7.1-8.0)^***^ | 10.2 (9.0-11.4)^***^ | 8.8 (8.2-9.5)^***^ |
| Time per DM/HTN new patient visit | 16.0 (9.9-22.0) | 11.0 (7.5-14.4) | 21.5 (0-129.5) | - |
| Time per DM/HTN follow-up patient visit | 10.3 (9.2-11.4)^***^ | 8.7 (8.0-9.4)^***^ | 10.0 (8.7-11.2) | 8.6 (7.8-9.4) |
| Number of patients observed^+^ |  |  |  |  |
| Average number of all patients seen | 6 (5-7) | 6 (4-7) | 2 (1-3) | 2 (1-3) |
| Average number of DM/HTN patients seen | 2 (1-2) | 2 (1-2) | 1 (0-1) | 1 (0-1) |
| Average number of non-DM/non-HTN patients seen | 4 (3-5) | 4 (3-5) | 1 (1-2) | 2 (1-2) |
| Number of patients observed (by facility, daily) |  |  |  |  |
| Average number of all patients seen | 13 (10-16) | 18 (13-22) | 4 (2.7) | 7 (4-10) |
| Average number of DM/HTN patients seen | 4 (1-6) | 6 (4-8) | 1 (0-3) | 2 (0-3) |
| Average number of non-DM/non-HTN patients seen | 9 (7-12) | 12 (8-16) | 3 (2-5) | 5 (3-7) |
| ***^***^p<0.001*. ^+^Patients observed per HCW in observed time; doesn’t represent all patient visits** | | | | |

| **Table S7. Modelled number of DM/HTN patient visits at TMS facilities by arm over two days** | | | | | | |
| --- | --- | --- | --- | --- | --- | --- |
|  | **SOC** | | | **Intervention** | | |
| **Time of patient visit** | **CMIS observed** | **TMS DM/HTN observed** | **TMS DM/HTN expected** | **CMIS observed** | **TMS DM/HTN observed** | **TMS DM/HTN expected** |
| 08:00–- 08:30 | 1423 | 1 | 42 | 2084 | 3 | 85 |
| 08:30–- 09:00 | 2713 | 9 | 80 | 3145 | 18 | 129 |
| 09:00–- 09:30 | 2402 | 13 | 71 | 2673 | 19 | 110 |
| 09:30–- 10:00 | 1734 | 15 | 51 | 1749 | 24 | 72 |
| 10:00–- 10:30 | 1442 | 16 | 43 | 1460 | 29 | 60 |
| 10:30–- 11:00 | 1069 | 13 | 32 | 995 | 14 | 41 |
| 11:00–- 11:30 | 806 | 24 | 24 | 854 | 35 | 35 |
| 11:30–- 12:00 | 772 | 15 | 23 | 592 | 15 | 24 |
| 12:00–- 12:30 | 570 | 15 | 17 | 458 | 14 | 19 |
| 12:30–- 13:00 | 349 | 10 | 10 | 304 | 11 | 13 |
| 13:00–- 13:30 | 254 | 7 | 8 | 149 | 6 | 6 |
| 13:30–- 14:00 | 191 | 2 | 6 | 90 | 4 | 4 |
| 14:00–- 14:30 | 261 | 0 | 8 | 247 | 3 | 10 |
| 14:30–- 15:00 | 184 | 3 | 5 | 214 | 8 | 9 |
| 15:00–- 15:30 | 136 | 0 | 4 | 131 | 0 | 5 |
| 15:30–- 16:00 | 56 | 0 | 2 | 89 | 0 | 4 |
| 16:00–- 16:30 | 20 | 0 | 1 | 31 | 0 | 1 |
| 16:30–- 17:00 | 12 | 0 | 0 | 17 | 0 | 1 |
| Total | 14394 | 143 | 427 | 15282 | 203 | 628 |


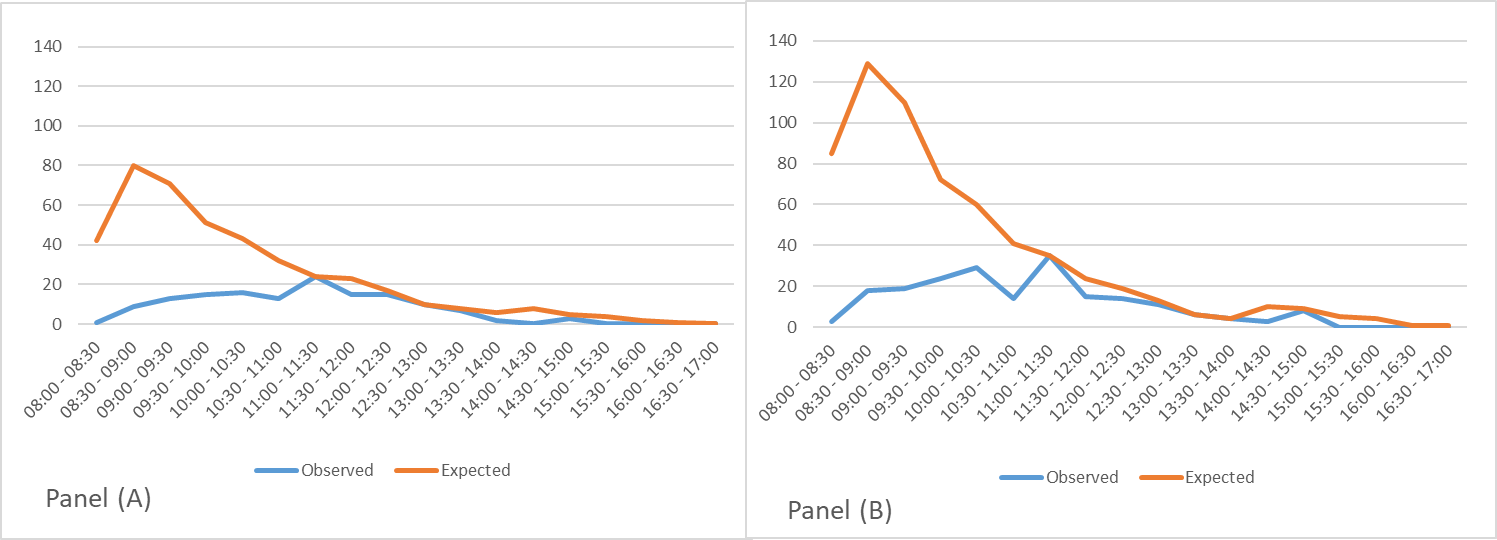


**Figure S3. Observed versus modelled DM/HTN patient visits;**

Panel (A): Patient visits at TMS control-arm facilities

Panel (B): Patient visits at TMS intervention-arm facilities

**Costing under two different disease prevalence scenarios**

To account for the uncertainty around the true prevalence of DM and HTN in Eswatini, we perform the bottom-up costing analysis for two more prevalence scenarios in addition to the high-prevalence scenario presented in the main manuscript. For these two scenarios, we assume a prevalence rate of 2.5% for DM and 22.5% for HTN as a low-prevalence scenario and that of DM 4.2% for DM and 38.3% for HTN as a medium-prevalence scenario. The estimated total costs are presented in Table S8.

| **Table S8. Costs of scale-up of WHO-PEN in Eswatini under alternative prevalence scenarios (in millions)** | | | |
| --- | --- | --- | --- |
| **Cost (in USD)** | **SOC (95% CI)** | **Intervention (95% CI)** | |
| **Low prevalence** | | | |
| Personnel | 2.76 (2.21-3.59) | | 1.57 (1.26-2.00) |
| *Salary costs* | 2.72 (2.17-3.53) | | 1.54 (1.24-1.96) |
| *Training costs* | 0.04 (0.03-0.06) | | 0.002 (0.002-0.003) |
| Medication | 0.20 (0.19-0.20) | | 0.20 (0.19-0.20) |
| Diagnostics | 0.06 (0.05-0.06) | | 0.05 (0.05-0.05) |
| Total cost | 3.03 (2.47-3.85) | | 1.83 (1.54-2.26) |
| **Medium prevalence** | | | |
| Personnel | 5.31 (4.25-6.90) | | 3.02 (2.43-3.84) |
| *Salary costs* | 5.23 (4.18-6.79) | | 2.97 (2.39-3.77) |
| *Training costs* | 0.08 (0.07-0.11) | | 0.04 (0.03-0.06) |
| Medication | 0.34 (0.32-0.35) | | 0.34 (0.32-0.35) |
| Diagnostics | 0.11 (0.11-0.12) | | 0.11 (0.11-0.12) |
| Total cost | 5.77 (4.70-7.36) | | 3.48 (2.88-4.29) |
